# (1,4,7,10,13,16-Hexaoxa­cyclo­octa­decane-κ^6^ *O*)bis­(tetra­hydro­furan-κ*O*)potassium bis­[(1,2,3,4-η)-anthracene]cobalt(−I) tetra­hydro­furan monosolvate

**DOI:** 10.1107/S1600536813032510

**Published:** 2013-12-07

**Authors:** Haiyan He, Wilhelm Klein, Thomas F. Fässler

**Affiliations:** a Technische Universität München, Department of Chemistry, Lichtenbergstrasse 4, 85747 Garching, Germany

## Abstract

The asymmetric unit of the title compound, [K(C_12_H_24_O_6_)(C_4_H_8_O)_2_][Co(C_14_H_10_)_2_]·C_4_H_8_O, consists of one cationic potassium complex, one anionic cobalt dianthracene complex and one tetra­hydro­furan solvent mol­ecule. The potassium cation is situated at the centre of an 18-crown-6 mol­ecule and between two tetra­hydro­furan mol­ecules, the latter coordin­ating above and below the mean plane formed by the O atoms of the crown ether mol­ecule. The Co atom is coordinated by eight C atoms of two anthracene mol­ecules in an η^4^ manner. The third free tetra­hydro­furan mol­ecule shows orientational disorder on two partially occupied positions [occupancy ratio 0.561 (8):0.439 (8)].

## Related literature   

For the synthesis, see: Brennessel, Young & Ellis (2002 ▶); Brennessel & Ellis (2012 ▶). Other homoleptic transition metal-anthracene complexes are reported by Elschenbroich *et al.* (1984 ▶); Brennessel, Ellis, Pomije *et al.* (2002 ▶), Brennessel, Ellis, Roush *et al.* (2002 ▶); Brennessel *et al.* (2007 ▶); Jilek *et al.* (2008 ▶). For related structures, see: Hanic & Mills (1968 ▶); Veauthier *et al.* (2000 ▶); Wang & Fässler (2009 ▶); Woolf *et al.* (2011 ▶); Zhu *et al.* (2006 ▶). Zintl compounds and their chemistry are reviewed by Scharfe *et al.* (2011 ▶). For a description of the Cambridge Structural database, see: Allen (2002 ▶).
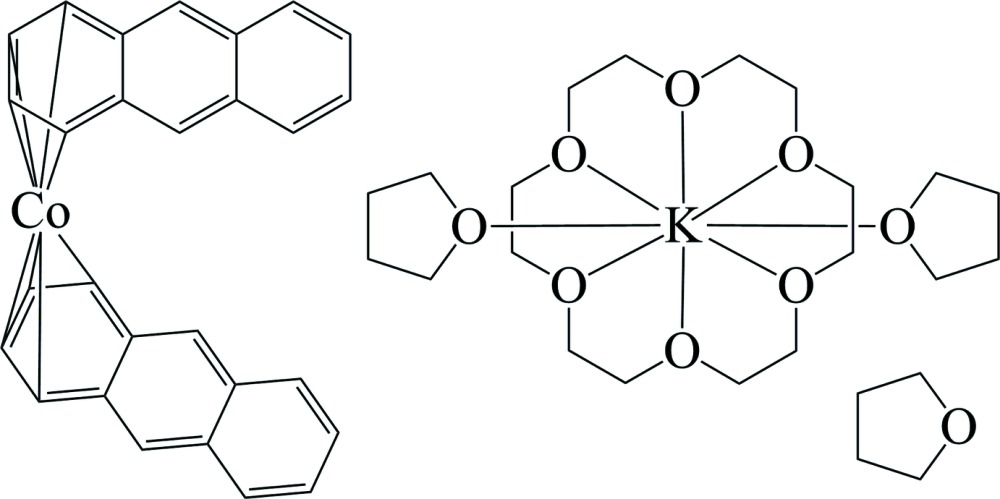


## Experimental   

### 

#### Crystal data  

[K(C_12_H_24_O_6_)(C_4_H_8_O)_2_][Co(C_14_H_10_)_2_]·C_4_H_8_O
*M* *_r_* = 935.09
Orthorhombic, 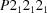

*a* = 13.0428 (10) Å
*b* = 13.3557 (10) Å
*c* = 27.4939 (17) Å
*V* = 4789.3 (6) Å^3^
*Z* = 4
Mo *K*α radiation
μ = 0.50 mm^−1^
*T* = 150 K
0.60 × 0.30 × 0.05 mm

#### Data collection  

Oxford Diffraction Xcalibur 3 diffractometer
Absorption correction: multi-scan (*CrysAlis RED*; Oxford Diffraction, 2009 ▶) *T*_min_ = 0.787, *T*_max_ = 0.975
62002 measured reflections
9364 independent reflections
4717 reflections with *I* > 2σ(*I*)
*R*_int_ = 0.105

#### Refinement  

*R*[*F* ^2^ > 2σ(*F* ^2^)] = 0.045
*wR*(*F* ^2^) = 0.076
*S* = 0.77
9364 reflections
604 parameters
H-atom parameters constrained
Δρ_max_ = 0.96 e Å^−3^
Δρ_min_ = −0.37 e Å^−3^
Absolute structure: Flack (1983 ▶), 4164 Friedel pairs
Absolute structure parameter: −0.005 (14)

### 

Data collection: *CrysAlis CCD* (Oxford Diffraction, 2009 ▶); cell refinement: *CrysAlis RED* (Oxford Diffraction, 2009 ▶); data reduction: *CrysAlis RED*; program(s) used to solve structure: *SHELXS97* (Sheldrick, 2008 ▶); program(s) used to refine structure: *SHELXL97* (Sheldrick, 2008 ▶); molecular graphics: *DIAMOND* (Brandenburg, 2012 ▶); software used to prepare material for publication: *SHELXL97*.

## Supplementary Material

Crystal structure: contains datablock(s) global, I. DOI: 10.1107/S1600536813032510/hg5364sup1.cif

Structure factors: contains datablock(s) I. DOI: 10.1107/S1600536813032510/hg5364Isup2.hkl

Additional supporting information:  crystallographic information; 3D view; checkCIF report

## Figures and Tables

**Fig. 1.**
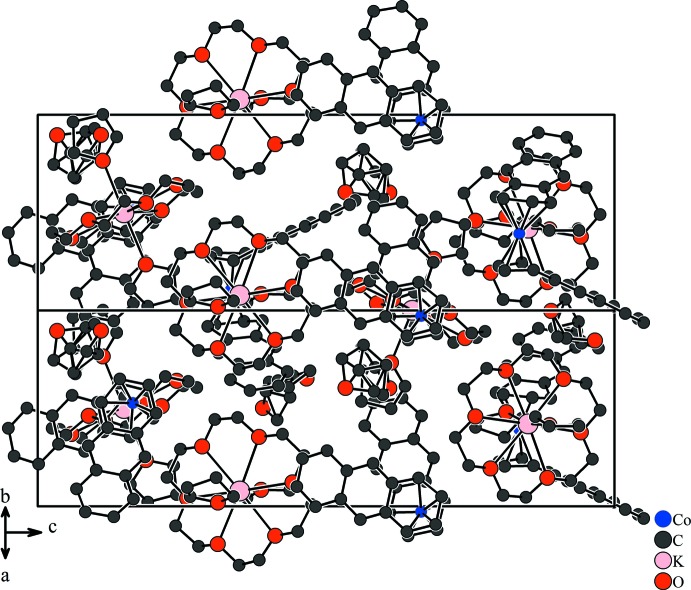
Unit cell of the title compound, view along [11 0].

**Fig. 2.**
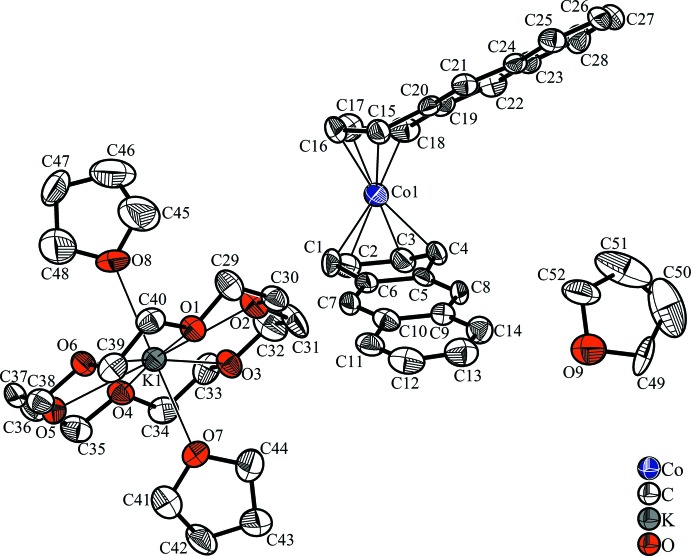
Asymmetric unit of the title compound. Displacement ellipsoids are drawn at the 50% probability level, H atoms and atoms of the disordered atoms of the free THF molecule are omitted for clarity.

## supplementary crystallographic information

### 1. Comment

Organometallic compounds with transition metals in a low oxidation state are important precursors for the formation of intermetalloid clusters from Zintl anions (Scharfe *et al.*, 2011). Transition metal complexes with polycyclic aromatic hydrocarbons as ligands can serve as storable, but reactive sources of low-valent metal atoms or even anions (Brennessel & Ellis, 2012). Within this group of compounds, anthracene has been found as a prominent, sometimes favoured ligand (Zhu *et al.*, 2006), which can act as a η^2^ (Woolf *et al.*, 2011), η^4^, and η^6^ (Hanic & Mills, 1968; Elschenbroich *et al.*, 1984), in its monohydrogenated form even as a η^5^ (Veauthier *et al.*, 2000) ligand. Regarding the entries of the Cambridge Structural Database (Allen, 2002), it is interesting that homoleptic anthracene complexes have been obtained exclusively with η^4^ coordination of the transition metals Ti, Zr, Hf (Jilek *et al.*, 2008), Nb (Brennessel, Ellis, Roush *et al.*, 2002), Ta (Brennessel, Ellis, Pomije *et al.*, 2002), Fe ((Brennessel *et al.*, 2007), and Co (Brennessel, Young & Ellis, 2002).

The title compound was obtained *via* reduction of CoBr_2_ by potassium anthracene in THF. Although the reaction conditions were very similar to those described by Brennessel, Young & Ellis (2002), we found the compound to crystallize including an additional free solvent molecule (Fig.1). Consequently different from the literature, our indexation resulted in the orthorhombic, but acentric space group *P*2_1_2_1_2_1_ instead of *P*1. Besides these differences, those structural entities, which are present in both compounds, show almost the same shape (Fig.2).

The Co atom is sandwiched by two anthracene molecules in 1,2,3,4-η^4^ coordination, respectively, with a slight tilt of 10.2° for the two planes formed by the respective coordinating atoms C1—C4. The C_6_ rings, which are directly bond to the Co atom, are in eclipsed position, but the main molecule axes form an angle of about 65°. The anthracene molecules are folded at an axis running through the C1 and C4 atoms, the angles between the planes formed by C1, C2, C3, and C4 and by C1, and C4—C14 are almost identical (29.18° and 28.99° for both molecules).

### 2. Experimental

To a deep-blue solution of K[C_14_H_10_] (3.43 mmol) in tetrahydrofuran (THF) (30 ml, -78°C) was added a bright-blue solution of CoBr_2_ (0.25 g, 1.15 mmol) in THF (30 ml, -78°C). The reaction was stirred overnight and warmed slowly to room temperature, the colour of the solution gradually changed to deep-pinkish-red. It was filtered to remove KBr, and 18-crown-6 (0.302 g, 1.15 mmol) in THF (10 ml) was added to the filtrate. Hexane (20 ml) was added and the volume was reduced to about 20 ml in vacuum. The brown-black needle-like product was crystallized at -40°C by diffusion of diethyl ether (50 ml) which yields the title compound (Yield: 41% based on CoBr_2_). The energy-dispersive X-ray analysis (EDX) shows an atomic ratio of Co/K close to 1:1 in all the analysed crystals of [K(C_12_H_24_O_6_)(C_4_H_8_O)_2_][Co(C_14_H_10_)_2_](C_4_H_8_O).

### 3. Refinement

The H atoms were included in calculated positions and treated as riding atoms: C—H = 0.93 Å with *U*_iso_(H) = 1.2 *U*_eq_ (parent C atom) for the aromatic, C—H = 0.97 Å with *U*_iso_(H) = 1.2 *U*_eq_ (parent C atom) for the crown ether, and C—H = 0.97 Å with *U*_iso_(H) = 1.5 *U*_eq_ (parent C atom) for the tetrahydrofuran H atoms. The free solvent molecule shows disorder and was refined at two positions with partial occupations of 56% and 44%, respectively. Two C atoms of the THF molecule with lower occupation have been refined with isotropic thermal displacement parameters.

### Crystal data

**Table:**

[K(C_12_H_24_O_6_)(C_4_H_8_O)_2_][Co(C_14_H_10_)_2_]·C_4_H_8_O	*F*(000) = 1992
*M**_r_* = 935.09	*D*_x_ = 1.297 Mg m^−^^3^
Orthorhombic, *P*2_1_2_1_2_1_	Mo *K*α radiation, λ = 0.71073 Å
Hall symbol: P 2ac 2ab	Cell parameters from 8368 reflections
*a* = 13.0428 (10) Å	θ = 3.0–37.0°
*b* = 13.3557 (10) Å	µ = 0.50 mm^−^^1^
*c* = 27.4939 (17) Å	*T* = 150 K
*V* = 4789.3 (6) Å^3^	Plate, brown-black
*Z* = 4	0.6 × 0.3 × 0.05 mm

### Data collection

**Table:**

Oxford Diffraction Xcalibur 3 diffractometer	9364 independent reflections
Radiation source: Enhance (Mo) X-ray Source	4717 reflections with *I* > 2σ(*I*)
Graphite monochromator	*R*_int_ = 0.105
Detector resolution: 16.0238 pixels mm^-1^	θ_max_ = 26.0°, θ_min_ = 3.0°
ω and φ scans	*h* = −16→16
Absorption correction: multi-scan (*CrysAlis RED*; Oxford Diffraction, 2009)	*k* = −16→16
*T*_min_ = 0.787, *T*_max_ = 0.975	*l* = −33→33
62002 measured reflections	

### Refinement

**Table:**

Refinement on *F*^2^	Secondary atom site location: difference Fourier map
Least-squares matrix: full	Hydrogen site location: inferred from neighbouring sites
*R*[*F*^2^ > 2σ(*F*^2^)] = 0.045	H-atom parameters constrained
*wR*(*F*^2^) = 0.076	*w* = 1/[σ^2^(*F*_o_^2^) + (0.0282*P*)^2^] where *P* = (*F*_o_^2^ + 2*F*_c_^2^)/3
*S* = 0.77	(Δ/σ)_max_ = 0.001
9364 reflections	Δρ_max_ = 0.96 e Å^−^^3^
604 parameters	Δρ_min_ = −0.37 e Å^−^^3^
0 restraints	Absolute structure: Flack (1983), 4164 Friedel pairs
Primary atom site location: structure-invariant direct methods	Absolute structure parameter: −0.005 (14)

### Special details

**Table:**

Experimental. Absorption correction: CrysAlis RED, Oxford Diffraction Ltd., Version 1.171.33.34d (release 27-02-2009 CrysAlis171 .NET) (compiled Feb 27 2009,15:38:38) Empirical absorption correction using spherical harmonics, implemented in SCALE3 ABSPACK scaling algorithm.
Geometry. All e.s.d.'s (except the e.s.d. in the dihedral angle between two l.s. planes) are estimated using the full covariance matrix. The cell e.s.d.'s are taken into account individually in the estimation of e.s.d.'s in distances, angles and torsion angles; correlations between e.s.d.'s in cell parameters are only used when they are defined by crystal symmetry. An approximate (isotropic) treatment of cell e.s.d.'s is used for estimating e.s.d.'s involving l.s. planes.
Refinement. Refinement of *F*^2^ against ALL reflections. The weighted *R*-factor *wR* and goodness of fit *S* are based on *F*^2^, conventional *R*-factors *R* are based on *F*, with *F* set to zero for negative *F*^2^. The threshold expression of *F*^2^ > σ(*F*^2^) is used only for calculating *R*-factors(gt) *etc*. and is not relevant to the choice of reflections for refinement. *R*-factors based on *F*^2^ are statistically about twice as large as those based on *F*, and *R*- factors based on ALL data will be even larger.

### Fractional atomic coordinates and isotropic or equivalent isotropic displacement parameters (Å^2^)

**Table:**

	*x*	*y*	*z*	*U*_iso_*/*U*_eq_	Occ. (<1)
Co1	0.53937 (4)	0.06723 (4)	0.164895 (18)	0.03905 (16)	
C1	0.4723 (3)	−0.0533 (3)	0.20524 (12)	0.0363 (10)	
H1A	0.4912	−0.0612	0.2395	0.044*	
C2	0.4052 (3)	0.0236 (3)	0.19268 (15)	0.0414 (11)	
H2A	0.3696	0.0632	0.2175	0.050*	
C3	0.3975 (3)	0.0471 (3)	0.14262 (14)	0.0406 (11)	
H3A	0.3554	0.1030	0.1310	0.049*	
C4	0.4585 (3)	−0.0103 (3)	0.11044 (13)	0.0340 (10)	
H4A	0.4654	0.0129	0.0768	0.041*	
C5	0.4841 (3)	−0.1143 (3)	0.12119 (13)	0.0312 (10)	
C6	0.4895 (3)	−0.1372 (3)	0.17228 (13)	0.0317 (10)	
C7	0.5148 (3)	−0.2325 (3)	0.18608 (13)	0.0357 (11)	
H7A	0.5188	−0.2475	0.2191	0.043*	
C8	0.5034 (3)	−0.1878 (3)	0.08783 (13)	0.0328 (11)	
H8A	0.4992	−0.1723	0.0549	0.039*	
C9	0.5291 (3)	−0.2855 (3)	0.10141 (14)	0.0347 (10)	
C10	0.5349 (3)	−0.3083 (3)	0.15194 (14)	0.0353 (10)	
C11	0.5544 (3)	−0.4078 (3)	0.16562 (16)	0.0463 (11)	
H11	0.5552	−0.4243	0.1985	0.056*	
C12	0.5723 (3)	−0.4807 (3)	0.13190 (18)	0.0546 (13)	
H12A	0.5846	−0.5463	0.1417	0.066*	
C13	0.5719 (3)	−0.4563 (4)	0.08267 (18)	0.0560 (14)	
H13A	0.5872	−0.5054	0.0598	0.067*	
C14	0.5495 (3)	−0.3612 (3)	0.06738 (15)	0.0433 (11)	
H14A	0.5478	−0.3467	0.0343	0.052*	
C15	0.7000 (3)	0.0552 (3)	0.16042 (13)	0.0359 (10)	
H15A	0.7317	−0.0100	0.1670	0.043*	
C16	0.6616 (3)	0.1113 (4)	0.20014 (14)	0.0471 (13)	
H16A	0.6741	0.0902	0.2338	0.056*	
C17	0.6016 (3)	0.1934 (4)	0.18996 (17)	0.0517 (13)	
H17A	0.5691	0.2330	0.2157	0.062*	
C18	0.5812 (3)	0.2105 (3)	0.14003 (16)	0.0434 (12)	
H18A	0.5263	0.2575	0.1316	0.052*	
C19	0.6563 (3)	0.1875 (3)	0.10353 (14)	0.0324 (11)	
C20	0.7222 (3)	0.1040 (3)	0.11462 (13)	0.0308 (10)	
C21	0.7949 (3)	0.0757 (3)	0.08149 (13)	0.0300 (10)	
H21A	0.8382	0.0225	0.0890	0.036*	
C22	0.6683 (3)	0.2359 (3)	0.05967 (15)	0.0375 (11)	
H22A	0.6258	0.2897	0.0524	0.045*	
C23	0.7429 (3)	0.2064 (3)	0.02550 (14)	0.0311 (11)	
C24	0.8064 (3)	0.1245 (3)	0.03617 (14)	0.0294 (10)	
C25	0.8818 (3)	0.0975 (3)	0.00161 (14)	0.0383 (11)	
H25A	0.9247	0.0435	0.0081	0.046*	
C26	0.8929 (4)	0.1485 (4)	−0.04079 (15)	0.0486 (13)	
H26A	0.9419	0.1279	−0.0632	0.058*	
C27	0.8327 (4)	0.2301 (4)	−0.05085 (16)	0.0530 (14)	
H27A	0.8427	0.2661	−0.0794	0.064*	
C28	0.7575 (4)	0.2585 (3)	−0.01869 (15)	0.0463 (12)	
H28	0.7156	0.3128	−0.0261	0.056*	
K1	0.03957 (7)	0.53903 (6)	0.14969 (3)	0.0335 (2)	
O1	−0.12066 (19)	0.4278 (2)	0.18781 (8)	0.0373 (7)	
O2	−0.0500 (2)	0.59109 (19)	0.24092 (9)	0.0443 (7)	
O3	0.1468 (2)	0.6590 (2)	0.21494 (9)	0.0417 (8)	
O4	0.2056 (2)	0.6488 (2)	0.11633 (9)	0.0398 (7)	
O5	0.1262 (2)	0.49597 (19)	0.05842 (8)	0.0378 (7)	
O6	−0.0561 (2)	0.40644 (18)	0.08827 (9)	0.0394 (7)	
C29	−0.1834 (3)	0.4796 (3)	0.22131 (14)	0.0486 (12)	
H29A	−0.2346	0.4346	0.2346	0.058*	
H29B	−0.2185	0.5342	0.2050	0.058*	
C30	−0.1184 (3)	0.5193 (3)	0.26089 (14)	0.0446 (12)	
H30A	−0.1609	0.5503	0.2856	0.054*	
H30B	−0.0800	0.4653	0.2759	0.054*	
C31	0.0203 (4)	0.6271 (3)	0.27595 (15)	0.0577 (13)	
H31A	0.0613	0.5722	0.2884	0.069*	
H31B	−0.0165	0.6570	0.3029	0.069*	
C32	0.0879 (4)	0.7032 (3)	0.25261 (16)	0.0653 (15)	
H32A	0.0463	0.7568	0.2393	0.078*	
H32B	0.1333	0.7318	0.2768	0.078*	
C33	0.2139 (3)	0.7294 (3)	0.19290 (15)	0.0485 (13)	
H33A	0.2603	0.7564	0.2172	0.058*	
H33B	0.1744	0.7843	0.1794	0.058*	
C34	0.2738 (3)	0.6798 (3)	0.15356 (15)	0.0459 (12)	
H34A	0.3239	0.7262	0.1404	0.055*	
H34B	0.3101	0.6223	0.1665	0.055*	
C35	0.2594 (3)	0.6084 (3)	0.07566 (15)	0.0487 (13)	
H35A	0.3005	0.5516	0.0858	0.058*	
H35B	0.3047	0.6587	0.0620	0.058*	
C36	0.1829 (3)	0.5760 (3)	0.03834 (13)	0.0429 (11)	
H36A	0.1376	0.6312	0.0302	0.051*	
H36B	0.2177	0.5545	0.0090	0.051*	
C37	0.0553 (3)	0.4539 (3)	0.02498 (12)	0.0390 (10)	
H37A	0.0910	0.4309	−0.0039	0.047*	
H37B	0.0053	0.5038	0.0153	0.047*	
C38	0.0032 (3)	0.3683 (3)	0.04914 (13)	0.0419 (11)	
H38A	−0.0408	0.3339	0.0261	0.050*	
H38B	0.0535	0.3210	0.0612	0.050*	
C39	−0.1043 (3)	0.3312 (3)	0.11580 (14)	0.0431 (11)	
H39A	−0.0533	0.2933	0.1337	0.052*	
H39B	−0.1405	0.2856	0.0944	0.052*	
C40	−0.1786 (3)	0.3792 (3)	0.15067 (15)	0.0447 (12)	
H40A	−0.2210	0.4275	0.1337	0.054*	
H40B	−0.2228	0.3287	0.1649	0.054*	
O7	0.1415 (3)	0.3777 (3)	0.18754 (11)	0.0738 (11)	
C41	0.2195 (4)	0.3313 (4)	0.16068 (19)	0.0842 (17)	
H41A	0.2307	0.3666	0.1303	0.101*	
H41B	0.2011	0.2625	0.1534	0.101*	
C42	0.3122 (4)	0.3344 (4)	0.19089 (17)	0.0776 (17)	
H42A	0.3499	0.3963	0.1861	0.093*	
H42B	0.3569	0.2781	0.1842	0.093*	
C43	0.2681 (4)	0.3285 (5)	0.24082 (18)	0.091 (2)	
H43A	0.2589	0.2595	0.2508	0.110*	
H43B	0.3117	0.3623	0.2642	0.110*	
C44	0.1701 (4)	0.3792 (4)	0.23591 (16)	0.0718 (16)	
H44A	0.1187	0.3456	0.2555	0.086*	
H44B	0.1760	0.4478	0.2471	0.086*	
O8	−0.0901 (3)	0.6752 (3)	0.11277 (14)	0.1021 (15)	
C45	−0.1710 (6)	0.7154 (5)	0.1368 (2)	0.147 (3)	
H45A	−0.2255	0.6662	0.1395	0.176*	
H45B	−0.1504	0.7345	0.1694	0.176*	
C46	−0.2049 (5)	0.7953 (5)	0.1129 (3)	0.105 (2)	
H46A	−0.1686	0.8548	0.1234	0.126*	
H46B	−0.2775	0.8049	0.1188	0.126*	
C47	−0.1865 (5)	0.7771 (4)	0.0613 (2)	0.097 (2)	
H47A	−0.1581	0.8357	0.0453	0.116*	
H47B	−0.2487	0.7568	0.0446	0.116*	
C48	−0.1109 (7)	0.6943 (6)	0.0635 (2)	0.195 (5)	
H48A	−0.1387	0.6348	0.0481	0.234*	
H48B	−0.0485	0.7131	0.0467	0.234*	
O9	0.1283 (5)	1.0242 (5)	0.0337 (2)	0.074 (3)	0.561 (8)
C49	0.1160 (9)	1.0277 (10)	0.0850 (4)	0.048 (4)	0.561 (8)
H49A	0.0442	1.0360	0.0934	0.072*	0.561 (8)
H49B	0.1542	1.0835	0.0985	0.072*	0.561 (8)
C50	0.1548 (17)	0.9331 (15)	0.1044 (5)	0.128 (7)	0.561 (8)
H50A	0.0989	0.8883	0.1126	0.192*	0.561 (8)
H50B	0.1962	0.9443	0.1333	0.192*	0.561 (8)
C51	0.218 (2)	0.892 (2)	0.0643 (13)	0.118 (12)	0.561 (8)
H51A	0.2861	0.8754	0.0757	0.177*	0.561 (8)
H51B	0.1864	0.8316	0.0512	0.177*	0.561 (8)
C52	0.2216 (7)	0.9687 (8)	0.0283 (4)	0.068 (3)	0.561 (8)
H52A	0.2262	0.9400	−0.0040	0.102*	0.561 (8)
H52B	0.2805	1.0117	0.0335	0.102*	0.561 (8)
O10	0.0540 (11)	0.9506 (9)	0.1099 (4)	0.112 (4)	0.439 (8)
C53	0.095 (2)	1.037 (3)	0.0972 (11)	0.169 (16)*	0.439 (8)
H53A	0.1287	1.0707	0.1241	0.254*	0.439 (8)
H53B	0.0461	1.0817	0.0818	0.254*	0.439 (8)
C54	0.1791 (13)	0.9867 (11)	0.0571 (6)	0.056 (4)*	0.439 (8)
H54A	0.1578	1.0026	0.0242	0.084*	0.439 (8)
H54B	0.2465	1.0155	0.0623	0.084*	0.439 (8)
C55	0.185 (2)	0.872 (3)	0.0633 (11)	0.066 (8)	0.439 (8)
H55A	0.2372	0.8511	0.0860	0.099*	0.439 (8)
H55B	0.1917	0.8368	0.0327	0.099*	0.439 (8)
C56	0.0744 (11)	0.8671 (12)	0.0848 (5)	0.094 (5)	0.439 (8)
H56A	0.0251	0.8596	0.0586	0.141*	0.439 (8)
H56B	0.0685	0.8093	0.1060	0.141*	0.439 (8)

### Atomic displacement parameters (Å^2^)

**Table:**

	*U*^11^	*U*^22^	*U*^33^	*U*^12^	*U*^13^	*U*^23^
Co1	0.0313 (3)	0.0510 (4)	0.0348 (3)	−0.0066 (3)	0.0057 (3)	−0.0050 (3)
C1	0.034 (3)	0.052 (3)	0.023 (2)	0.003 (3)	0.0025 (19)	−0.007 (2)
C2	0.042 (3)	0.041 (3)	0.041 (3)	−0.009 (2)	0.006 (2)	−0.003 (2)
C3	0.042 (3)	0.039 (3)	0.040 (3)	−0.001 (2)	0.003 (2)	0.007 (2)
C4	0.029 (3)	0.044 (3)	0.028 (2)	−0.007 (3)	−0.002 (2)	0.011 (2)
C5	0.019 (3)	0.047 (3)	0.028 (2)	−0.008 (2)	0.0011 (18)	0.008 (2)
C6	0.021 (2)	0.044 (3)	0.030 (2)	−0.0082 (19)	−0.0003 (18)	0.005 (2)
C7	0.028 (3)	0.051 (3)	0.028 (2)	−0.008 (2)	−0.0022 (19)	0.005 (2)
C8	0.030 (3)	0.047 (3)	0.021 (2)	−0.005 (2)	0.0027 (18)	0.001 (2)
C9	0.031 (3)	0.038 (3)	0.035 (3)	−0.006 (2)	0.001 (2)	0.001 (2)
C10	0.020 (2)	0.039 (3)	0.047 (3)	0.002 (2)	0.000 (2)	0.015 (2)
C11	0.028 (3)	0.057 (3)	0.054 (3)	−0.008 (2)	0.000 (2)	0.013 (3)
C12	0.038 (3)	0.042 (3)	0.084 (4)	0.005 (2)	0.004 (3)	0.013 (3)
C13	0.042 (3)	0.056 (4)	0.069 (3)	−0.001 (3)	0.012 (2)	−0.012 (3)
C14	0.041 (3)	0.045 (3)	0.043 (3)	0.002 (3)	0.002 (2)	0.001 (2)
C15	0.032 (2)	0.040 (3)	0.035 (2)	−0.002 (2)	−0.002 (2)	0.003 (2)
C16	0.040 (3)	0.073 (4)	0.029 (3)	−0.010 (3)	0.007 (2)	0.006 (2)
C17	0.035 (3)	0.073 (4)	0.047 (3)	−0.002 (3)	0.012 (2)	−0.023 (3)
C18	0.026 (3)	0.044 (3)	0.060 (3)	0.001 (2)	0.007 (2)	−0.010 (2)
C19	0.035 (3)	0.030 (3)	0.033 (3)	−0.009 (2)	0.001 (2)	−0.011 (2)
C20	0.022 (2)	0.039 (3)	0.031 (2)	−0.006 (2)	−0.0011 (19)	−0.006 (2)
C21	0.020 (2)	0.027 (2)	0.044 (2)	0.004 (2)	0.0037 (19)	−0.006 (2)
C22	0.035 (3)	0.026 (3)	0.051 (3)	0.005 (2)	−0.005 (2)	−0.001 (2)
C23	0.034 (3)	0.028 (3)	0.031 (3)	−0.006 (2)	0.001 (2)	−0.004 (2)
C24	0.028 (3)	0.027 (3)	0.034 (2)	−0.008 (2)	0.004 (2)	−0.005 (2)
C25	0.036 (3)	0.032 (3)	0.047 (3)	−0.001 (2)	0.004 (2)	−0.002 (2)
C26	0.053 (3)	0.058 (3)	0.035 (3)	−0.022 (3)	0.015 (2)	−0.006 (3)
C27	0.069 (4)	0.057 (4)	0.033 (3)	−0.025 (3)	0.006 (3)	0.012 (3)
C28	0.059 (4)	0.040 (3)	0.040 (3)	0.000 (3)	−0.015 (3)	0.009 (2)
K1	0.0288 (5)	0.0366 (5)	0.0352 (5)	−0.0008 (5)	−0.0023 (4)	−0.0025 (4)
O1	0.0298 (17)	0.0412 (17)	0.0408 (15)	−0.0051 (15)	0.0016 (14)	−0.0076 (15)
O2	0.0363 (19)	0.0461 (19)	0.0506 (17)	−0.0129 (17)	0.0057 (16)	−0.0120 (14)
O3	0.042 (2)	0.041 (2)	0.0417 (18)	−0.0060 (16)	0.0012 (15)	−0.0150 (15)
O4	0.0295 (19)	0.0456 (19)	0.0441 (18)	−0.0011 (15)	0.0018 (15)	−0.0061 (15)
O5	0.0342 (18)	0.0434 (18)	0.0357 (15)	−0.0048 (15)	0.0004 (14)	0.0001 (14)
O6	0.0381 (19)	0.0425 (18)	0.0375 (16)	0.0005 (16)	0.0042 (14)	−0.0034 (14)
C29	0.048 (3)	0.049 (3)	0.049 (3)	−0.009 (3)	0.017 (3)	0.001 (2)
C30	0.034 (3)	0.051 (3)	0.049 (3)	0.000 (3)	0.005 (2)	−0.012 (2)
C31	0.051 (3)	0.073 (3)	0.049 (3)	−0.012 (3)	0.017 (3)	−0.034 (3)
C32	0.052 (4)	0.074 (4)	0.070 (4)	−0.007 (3)	−0.002 (3)	−0.037 (3)
C33	0.041 (3)	0.052 (3)	0.052 (3)	−0.018 (3)	−0.015 (3)	0.002 (3)
C34	0.034 (3)	0.047 (3)	0.057 (3)	−0.008 (2)	−0.001 (3)	−0.004 (3)
C35	0.034 (3)	0.054 (3)	0.059 (3)	−0.005 (2)	0.017 (2)	0.002 (2)
C36	0.043 (3)	0.046 (3)	0.039 (2)	0.002 (3)	0.014 (2)	0.005 (2)
C37	0.040 (3)	0.045 (3)	0.031 (2)	0.006 (2)	0.002 (2)	−0.005 (2)
C38	0.032 (3)	0.044 (3)	0.049 (3)	0.000 (2)	−0.007 (2)	−0.009 (2)
C39	0.036 (3)	0.041 (3)	0.052 (3)	−0.013 (2)	−0.008 (2)	−0.010 (3)
C40	0.028 (3)	0.050 (3)	0.055 (3)	−0.006 (2)	−0.002 (2)	0.008 (2)
O7	0.066 (3)	0.109 (3)	0.046 (2)	0.044 (2)	−0.0049 (19)	0.0008 (19)
C41	0.093 (5)	0.103 (5)	0.056 (3)	0.030 (4)	0.005 (4)	−0.007 (4)
C42	0.045 (4)	0.130 (5)	0.058 (3)	0.021 (3)	0.001 (3)	−0.034 (4)
C43	0.072 (5)	0.139 (6)	0.063 (4)	0.047 (4)	−0.010 (3)	−0.012 (4)
C44	0.068 (4)	0.100 (4)	0.048 (3)	0.011 (3)	−0.007 (3)	0.005 (3)
O8	0.105 (3)	0.132 (3)	0.069 (3)	0.091 (3)	0.007 (2)	0.018 (3)
C45	0.210 (9)	0.127 (7)	0.104 (6)	0.107 (6)	0.051 (6)	0.044 (5)
C46	0.086 (5)	0.091 (5)	0.138 (7)	0.039 (4)	0.013 (5)	0.004 (5)
C47	0.152 (7)	0.091 (5)	0.049 (4)	0.045 (4)	−0.031 (4)	0.007 (3)
C48	0.290 (11)	0.207 (9)	0.088 (6)	0.195 (8)	0.016 (6)	0.016 (5)
O9	0.074 (6)	0.076 (5)	0.072 (5)	0.019 (4)	−0.012 (4)	−0.008 (4)
C49	0.048 (7)	0.058 (8)	0.037 (6)	−0.015 (6)	0.028 (5)	−0.006 (6)
C50	0.148 (19)	0.148 (17)	0.088 (11)	−0.058 (15)	−0.015 (11)	0.071 (12)
C51	0.10 (2)	0.035 (11)	0.22 (2)	−0.012 (13)	−0.087 (19)	−0.002 (13)
C52	0.056 (8)	0.058 (8)	0.091 (8)	0.022 (6)	−0.034 (6)	−0.015 (7)
O10	0.106 (11)	0.084 (8)	0.146 (9)	0.006 (8)	0.012 (8)	−0.025 (7)
C55	0.067 (18)	0.07 (2)	0.059 (12)	−0.014 (12)	−0.012 (10)	0.020 (10)
C56	0.059 (12)	0.105 (14)	0.119 (12)	−0.023 (10)	0.007 (9)	−0.018 (10)

### Geometric parameters (Å, º)

**Table:**

Co1—C1	2.142 (4)	C29—H29A	0.9700
Co1—C2	1.997 (4)	C29—H29B	0.9700
Co1—C3	1.967 (4)	C30—H30A	0.9700
Co1—C4	2.104 (4)	C30—H30B	0.9700
Co1—C15	2.105 (4)	C31—C32	1.491 (5)
Co1—C16	1.956 (4)	C31—H31A	0.9700
Co1—C17	1.993 (4)	C31—H31B	0.9700
Co1—C18	2.103 (4)	C32—H32A	0.9700
C1—C2	1.393 (5)	C32—H32B	0.9700
C1—C6	1.458 (5)	C33—C34	1.490 (5)
C1—H1A	0.9800	C33—H33A	0.9700
C2—C3	1.415 (5)	C33—H33B	0.9700
C2—H2A	0.9800	C34—H34A	0.9700
C3—C4	1.415 (5)	C34—H34B	0.9700
C3—H3A	0.9800	C35—C36	1.495 (5)
C4—C5	1.459 (5)	C35—H35A	0.9700
C4—H4A	0.9800	C35—H35B	0.9700
C5—C8	1.367 (5)	C36—H36A	0.9700
C5—C6	1.439 (5)	C36—H36B	0.9700
C6—C7	1.368 (5)	C37—C38	1.487 (4)
C7—C10	1.406 (5)	C37—H37A	0.9700
C7—H7A	0.9300	C37—H37B	0.9700
C8—C9	1.398 (5)	C38—H38A	0.9700
C8—H8A	0.9300	C38—H38B	0.9700
C9—C14	1.402 (5)	C39—C40	1.506 (5)
C9—C10	1.424 (5)	C39—H39A	0.9700
C10—C11	1.404 (5)	C39—H39B	0.9700
C11—C12	1.365 (5)	C40—H40A	0.9700
C11—H11	0.9300	C40—H40B	0.9700
C12—C13	1.392 (5)	O7—C44	1.382 (4)
C12—H12A	0.9300	O7—C41	1.401 (5)
C13—C14	1.370 (5)	C41—C42	1.468 (6)
C13—H13A	0.9300	C41—H41A	0.9700
C14—H14A	0.9300	C41—H41B	0.9700
C15—C16	1.416 (5)	C42—C43	1.490 (6)
C15—C20	1.448 (5)	C42—H42A	0.9700
C15—H15A	0.9800	C42—H42B	0.9700
C16—C17	1.375 (5)	C43—C44	1.453 (6)
C16—H16A	0.9800	C43—H43A	0.9700
C17—C18	1.417 (5)	C43—H43B	0.9700
C17—H17A	0.9800	C44—H44A	0.9700
C18—C19	1.436 (5)	C44—H44B	0.9700
C18—H18A	0.9800	O8—C45	1.356 (6)
C19—C22	1.377 (5)	O8—C48	1.405 (6)
C19—C20	1.441 (5)	C45—C46	1.329 (6)
C20—C21	1.368 (4)	C45—H45A	0.9700
C21—C24	1.414 (5)	C45—H45B	0.9700
C21—H21A	0.9300	C46—C47	1.460 (6)
C22—C23	1.409 (5)	C46—H46A	0.9700
C22—H22A	0.9300	C46—H46B	0.9700
C23—C24	1.403 (5)	C47—C48	1.482 (7)
C23—C28	1.413 (5)	C47—H47A	0.9700
C24—C25	1.414 (5)	C47—H47B	0.9700
C25—C26	1.358 (5)	C48—H48A	0.9700
C25—H25A	0.9300	C48—H48B	0.9700
C26—C27	1.372 (6)	O9—C49	1.422 (13)
C26—H26A	0.9300	O9—C52	1.433 (11)
C27—C28	1.374 (6)	C49—C50	1.46 (2)
C27—H27A	0.9300	C49—H49A	0.9700
C28—H28	0.9300	C49—H49B	0.9700
K1—O8	2.683 (3)	C50—C51	1.48 (4)
K1—O7	2.738 (3)	C50—H50A	0.9700
K1—O6	2.746 (2)	C50—H50B	0.9700
K1—O1	2.770 (3)	C51—C52	1.43 (3)
K1—O4	2.771 (3)	C51—H51A	0.9700
K1—O3	2.782 (3)	C51—H51B	0.9700
K1—O5	2.811 (2)	C52—H52A	0.9700
K1—O2	2.853 (3)	C52—H52B	0.9700
O1—C40	1.426 (4)	O10—C53	1.32 (3)
O1—C29	1.413 (4)	O10—C56	1.340 (16)
O2—C30	1.420 (4)	C53—C54	1.69 (3)
O2—C31	1.414 (4)	C53—H53A	0.9700
O3—C32	1.418 (4)	C53—H53B	0.9700
O3—C33	1.420 (4)	C54—C55	1.54 (4)
O4—C34	1.419 (4)	C54—H54A	0.9700
O4—C35	1.426 (4)	C54—H54B	0.9700
O5—C36	1.413 (4)	C55—C56	1.56 (3)
O5—C37	1.419 (4)	C55—H55A	0.9700
O6—C38	1.419 (4)	C55—H55B	0.9700
O6—C39	1.406 (4)	C56—H56A	0.9700
C29—C30	1.477 (5)	C56—H56B	0.9700
			
C16—Co1—C3	164.10 (19)	C36—O5—K1	113.8 (2)
C16—Co1—C17	40.74 (16)	C37—O5—K1	113.4 (2)
C3—Co1—C17	127.3 (2)	C39—O6—C38	113.3 (3)
C16—Co1—C2	127.78 (16)	C39—O6—K1	109.4 (2)
C3—Co1—C2	41.83 (14)	C38—O6—K1	116.8 (2)
C17—Co1—C2	118.14 (17)	O1—C29—C30	108.9 (3)
C16—Co1—C18	71.10 (17)	O1—C29—H29A	109.9
C3—Co1—C18	105.48 (17)	C30—C29—H29A	109.9
C17—Co1—C18	40.36 (15)	O1—C29—H29B	109.9
C2—Co1—C18	128.11 (17)	C30—C29—H29B	109.9
C16—Co1—C4	155.38 (18)	H29A—C29—H29B	108.3
C3—Co1—C4	40.52 (14)	O2—C30—C29	108.5 (3)
C17—Co1—C4	150.03 (18)	O2—C30—H30A	110.0
C2—Co1—C4	71.89 (16)	C29—C30—H30A	110.0
C18—Co1—C4	110.26 (16)	O2—C30—H30B	110.0
C16—Co1—C15	40.61 (15)	C29—C30—H30B	110.0
C3—Co1—C15	155.14 (16)	H30A—C30—H30B	108.4
C17—Co1—C15	71.30 (16)	O2—C31—C32	108.8 (4)
C2—Co1—C15	150.74 (17)	O2—C31—H31A	109.9
C18—Co1—C15	78.04 (16)	C32—C31—H31A	109.9
C4—Co1—C15	114.81 (15)	O2—C31—H31B	109.9
C16—Co1—C1	107.61 (16)	C32—C31—H31B	109.9
C3—Co1—C1	70.99 (15)	H31A—C31—H31B	108.3
C17—Co1—C1	128.51 (17)	O3—C32—C31	110.5 (3)
C2—Co1—C1	39.14 (14)	O3—C32—H32A	109.5
C18—Co1—C1	163.30 (15)	C31—C32—H32A	109.5
C4—Co1—C1	78.10 (13)	O3—C32—H32B	109.5
C15—Co1—C1	112.28 (16)	C31—C32—H32B	109.5
C2—C1—C6	120.6 (3)	H32A—C32—H32B	108.1
C2—C1—Co1	64.8 (2)	O3—C33—C34	109.8 (3)
C6—C1—Co1	101.1 (2)	O3—C33—H33A	109.7
C2—C1—H1A	118.4	C34—C33—H33A	109.7
C6—C1—H1A	118.4	O3—C33—H33B	109.7
Co1—C1—H1A	118.4	C34—C33—H33B	109.7
C1—C2—C3	116.7 (4)	H33A—C33—H33B	108.2
C1—C2—Co1	76.1 (3)	O4—C34—C33	108.9 (3)
C3—C2—Co1	68.0 (2)	O4—C34—H34A	109.9
C1—C2—H2A	121.6	C33—C34—H34A	109.9
C3—C2—H2A	121.6	O4—C34—H34B	109.9
Co1—C2—H2A	121.6	C33—C34—H34B	109.9
C4—C3—C2	116.6 (4)	H34A—C34—H34B	108.3
C4—C3—Co1	74.9 (2)	O4—C35—C36	108.6 (3)
C2—C3—Co1	70.2 (2)	O4—C35—H35A	110.0
C4—C3—H3A	121.6	C36—C35—H35A	110.0
C2—C3—H3A	121.6	O4—C35—H35B	110.0
Co1—C3—H3A	121.6	C36—C35—H35B	110.0
C3—C4—C5	121.2 (3)	H35A—C35—H35B	108.4
C3—C4—Co1	64.6 (2)	O5—C36—C35	107.5 (3)
C5—C4—Co1	102.1 (2)	O5—C36—H36A	110.2
C3—C4—H4A	118.1	C35—C36—H36A	110.2
C5—C4—H4A	118.1	O5—C36—H36B	110.2
Co1—C4—H4A	118.1	C35—C36—H36B	110.2
C8—C5—C6	119.5 (4)	H36A—C36—H36B	108.5
C8—C5—C4	126.2 (3)	O5—C37—C38	108.2 (3)
C6—C5—C4	114.3 (3)	O5—C37—H37A	110.1
C7—C6—C5	118.7 (3)	C38—C37—H37A	110.1
C7—C6—C1	125.4 (3)	O5—C37—H37B	110.1
C5—C6—C1	115.8 (4)	C38—C37—H37B	110.1
C6—C7—C10	122.0 (3)	H37A—C37—H37B	108.4
C6—C7—H7A	119.0	O6—C38—C37	108.2 (3)
C10—C7—H7A	119.0	O6—C38—H38A	110.1
C5—C8—C9	122.4 (3)	C37—C38—H38A	110.1
C5—C8—H8A	118.8	O6—C38—H38B	110.1
C9—C8—H8A	118.8	C37—C38—H38B	110.1
C8—C9—C14	122.6 (3)	H38A—C38—H38B	108.4
C8—C9—C10	118.2 (3)	O6—C39—C40	109.1 (3)
C14—C9—C10	119.2 (4)	O6—C39—K1	48.14 (16)
C11—C10—C7	122.4 (4)	C40—C39—K1	80.6 (2)
C11—C10—C9	118.2 (4)	O6—C39—H39A	109.9
C7—C10—C9	119.2 (3)	C40—C39—H39A	109.9
C12—C11—C10	121.6 (4)	K1—C39—H39A	84.9
C12—C11—H11	119.2	O6—C39—H39B	109.9
C10—C11—H11	119.2	C40—C39—H39B	109.9
C11—C12—C13	119.5 (4)	K1—C39—H39B	157.9
C11—C12—H12A	120.3	H39A—C39—H39B	108.3
C13—C12—H12A	120.3	O1—C40—C39	108.0 (3)
C14—C13—C12	121.1 (4)	O1—C40—H40A	110.1
C14—C13—H13A	119.5	C39—C40—H40A	110.1
C12—C13—H13A	119.5	O1—C40—H40B	110.1
C13—C14—C9	120.2 (4)	C39—C40—H40B	110.1
C13—C14—H14A	119.9	H40A—C40—H40B	108.4
C9—C14—H14A	119.9	C44—O7—C41	108.5 (4)
C16—C15—C20	120.2 (4)	C44—O7—K1	119.1 (3)
C16—C15—Co1	64.0 (2)	C41—O7—K1	120.0 (3)
C20—C15—Co1	102.5 (2)	O7—C41—C42	106.7 (4)
C16—C15—H15A	118.5	O7—C41—H41A	110.4
C20—C15—H15A	118.5	C42—C41—H41A	110.4
Co1—C15—H15A	118.5	O7—C41—H41B	110.4
C17—C16—C15	117.8 (4)	C42—C41—H41B	110.4
C17—C16—Co1	71.1 (3)	H41A—C41—H41B	108.6
C15—C16—Co1	75.3 (2)	C41—C42—C43	101.6 (4)
C17—C16—H16A	121.1	C41—C42—H42A	111.4
C15—C16—H16A	121.1	C43—C42—H42A	111.4
Co1—C16—H16A	121.1	C41—C42—H42B	111.4
C16—C17—C18	115.7 (4)	C43—C42—H42B	111.4
C16—C17—Co1	68.2 (3)	H42A—C42—H42B	109.3
C18—C17—Co1	74.0 (3)	C44—C43—C42	103.2 (4)
C16—C17—H17A	122.0	C44—C43—H43A	111.1
C18—C17—H17A	122.0	C42—C43—H43A	111.1
Co1—C17—H17A	122.0	C44—C43—H43B	111.1
C17—C18—C19	120.9 (4)	C42—C43—H43B	111.1
C17—C18—Co1	65.6 (3)	H43A—C43—H43B	109.1
C19—C18—Co1	102.1 (3)	O7—C44—C43	108.7 (4)
C17—C18—H18A	118.0	O7—C44—H44A	110.0
C19—C18—H18A	118.0	C43—C44—H44A	110.0
Co1—C18—H18A	118.0	O7—C44—H44B	110.0
C22—C19—C18	126.1 (4)	C43—C44—H44B	110.0
C22—C19—C20	118.7 (4)	H44A—C44—H44B	108.3
C18—C19—C20	115.1 (4)	C45—O8—C48	104.4 (5)
C21—C20—C19	119.1 (4)	C45—O8—K1	125.1 (4)
C21—C20—C15	126.4 (4)	C48—O8—K1	127.6 (4)
C19—C20—C15	114.4 (3)	C46—C45—O8	109.6 (6)
C20—C21—C24	122.2 (4)	C46—C45—H45A	109.8
C20—C21—H21A	118.9	O8—C45—H45A	109.8
C24—C21—H21A	118.9	C46—C45—H45B	109.8
C19—C22—C23	122.1 (4)	O8—C45—H45B	109.8
C19—C22—H22A	118.9	H45A—C45—H45B	108.2
C23—C22—H22A	118.9	C45—C46—C47	107.0 (5)
C24—C23—C22	119.1 (4)	C45—C46—H46A	110.3
C24—C23—C28	119.0 (4)	C47—C46—H46A	110.3
C22—C23—C28	121.9 (4)	C45—C46—H46B	110.3
C23—C24—C25	118.0 (4)	C47—C46—H46B	110.3
C23—C24—C21	118.8 (4)	H46A—C46—H46B	108.6
C25—C24—C21	123.2 (4)	C46—C47—C48	101.1 (5)
C26—C25—C24	121.5 (4)	C46—C47—H47A	111.5
C26—C25—H25A	119.2	C48—C47—H47A	111.5
C24—C25—H25A	119.2	C46—C47—H47B	111.5
C25—C26—C27	120.7 (4)	C48—C47—H47B	111.5
C25—C26—H26A	119.7	H47A—C47—H47B	109.4
C27—C26—H26A	119.7	O8—C48—C47	107.7 (5)
C26—C27—C28	119.9 (4)	O8—C48—H48A	110.2
C26—C27—H27A	120.0	C47—C48—H48A	110.2
C28—C27—H27A	120.0	O8—C48—H48B	110.2
C27—C28—C23	120.9 (4)	C47—C48—H48B	110.2
C27—C28—H28	119.6	H48A—C48—H48B	108.5
C23—C28—H28	119.6	C49—O9—C52	102.5 (8)
O8—K1—O7	169.58 (13)	O9—C49—C50	107.1 (12)
O8—K1—O6	85.30 (11)	O9—C49—H49A	110.3
O7—K1—O6	86.94 (9)	C50—C49—H49A	110.3
O8—K1—O1	91.78 (11)	O9—C49—H49B	110.3
O7—K1—O1	78.47 (9)	C50—C49—H49B	110.3
O6—K1—O1	62.88 (8)	H49A—C49—H49B	108.5
O8—K1—O4	90.50 (11)	C49—C50—C51	104.1 (17)
O7—K1—O4	99.38 (9)	C49—C50—H50A	110.9
O6—K1—O4	119.50 (8)	C51—C50—H50A	110.9
O1—K1—O4	176.85 (8)	C49—C50—H50B	110.9
O8—K1—O3	99.82 (11)	C51—C50—H50B	110.9
O7—K1—O3	87.94 (9)	H50A—C50—H50B	109.0
O6—K1—O3	174.87 (8)	C52—C51—C50	105 (2)
O1—K1—O3	116.37 (8)	C52—C51—H51A	110.7
O4—K1—O3	61.05 (8)	C50—C51—H51A	110.7
O8—K1—O5	93.11 (10)	C52—C51—H51B	110.7
O7—K1—O5	89.04 (9)	C50—C51—H51B	110.7
O6—K1—O5	60.12 (8)	H51A—C51—H51B	108.8
O1—K1—O5	122.08 (8)	C51—C52—O9	105.7 (17)
O4—K1—O5	59.92 (8)	C51—C52—H52A	110.6
O3—K1—O5	119.43 (9)	O9—C52—H52A	110.6
O8—K1—O2	84.79 (10)	C51—C52—H52B	110.6
O7—K1—O2	93.24 (9)	O9—C52—H52B	110.6
O6—K1—O2	120.77 (9)	H52A—C52—H52B	108.7
O1—K1—O2	59.27 (8)	C53—O10—C56	120.7 (16)
O4—K1—O2	118.82 (8)	O10—C53—C54	95.1 (19)
O3—K1—O2	59.92 (8)	O10—C53—H53A	112.7
O5—K1—O2	177.58 (8)	C54—C53—H53A	112.7
O8—K1—C39	95.71 (12)	O10—C53—H53B	112.7
O7—K1—C39	74.66 (11)	C54—C53—H53B	112.7
O6—K1—C39	22.42 (7)	H53A—C53—H53B	110.2
O1—K1—C39	42.80 (8)	C55—C54—C53	111 (2)
O4—K1—C39	139.04 (9)	C55—C54—H54A	109.5
O3—K1—C39	154.63 (9)	C53—C54—H54A	109.5
O5—K1—C39	79.30 (9)	C55—C54—H54B	109.5
O2—K1—C39	102.07 (9)	C53—C54—H54B	109.5
C29—O1—C40	112.5 (3)	H54A—C54—H54B	108.1
C29—O1—K1	114.9 (2)	C54—C55—C56	92 (2)
C40—O1—K1	111.9 (2)	C54—C55—H55A	113.2
C31—O2—C30	111.9 (3)	C56—C55—H55A	113.2
C31—O2—K1	114.6 (2)	C54—C55—H55B	113.2
C30—O2—K1	115.6 (2)	C56—C55—H55B	113.2
C32—O3—C33	111.7 (3)	H55A—C55—H55B	110.6
C32—O3—K1	116.0 (2)	O10—C56—C55	110.1 (16)
C33—O3—K1	114.6 (2)	O10—C56—H56A	109.7
C34—O4—C35	111.5 (3)	C55—C56—H56A	109.7
C34—O4—K1	114.0 (2)	O10—C56—H56B	109.7
C35—O4—K1	116.4 (2)	C55—C56—H56B	109.7
C36—O5—C37	112.8 (3)	H56A—C56—H56B	108.2
			
C1—C2—C3—C4	0.3 (6)	C18—C19—C22—C23	−178.4 (4)
C6—C1—C2—C3	−32.1 (5)	C20—C19—C22—C23	−0.1 (6)
C2—C3—C4—C5	30.9 (6)	C19—C22—C23—C24	0.7 (6)
Co1—C1—C2—C3	56.2 (3)	C19—C22—C23—C28	−177.6 (4)
Co1—C2—C3—C4	60.7 (3)	C22—C23—C24—C25	−179.0 (4)
C1—C2—C3—Co1	−60.4 (3)	C28—C23—C24—C25	−0.6 (5)
C2—C3—C4—Co1	−58.2 (3)	C22—C23—C24—C21	−1.5 (5)
C6—C1—C2—Co1	−88.2 (3)	C28—C23—C24—C21	176.9 (4)
Co1—C3—C4—C5	89.1 (4)	C20—C21—C24—C23	1.8 (6)
C2—C1—C6—C5	32.8 (5)	C20—C21—C24—C25	179.2 (4)
C3—C4—C5—C6	−29.3 (5)	C23—C24—C25—C26	−0.1 (6)
C2—C1—C6—C7	−150.6 (4)	C21—C24—C25—C26	−177.6 (4)
C3—C4—C5—C8	151.3 (4)	C24—C25—C26—C27	1.7 (6)
C4—C5—C6—C1	−2.0 (5)	C25—C26—C27—C28	−2.5 (7)
C8—C5—C6—C7	0.6 (5)	C26—C27—C28—C23	1.8 (7)
C4—C5—C6—C7	−178.9 (3)	C24—C23—C28—C27	−0.2 (6)
C8—C5—C6—C1	177.4 (3)	C22—C23—C28—C27	178.2 (4)
C5—C6—C7—C10	−0.4 (5)	C40—O1—C29—C30	174.6 (3)
C1—C6—C7—C10	−177.0 (4)	C31—O2—C30—C29	−175.5 (3)
C6—C5—C8—C9	−0.5 (6)	O1—C29—C30—O2	64.4 (4)
C4—C5—C8—C9	178.9 (4)	C30—O2—C31—C32	−178.9 (3)
C5—C8—C9—C14	−179.6 (4)	C33—O3—C32—C31	−178.5 (4)
C5—C8—C9—C10	0.2 (6)	O2—C31—C32—O3	−63.1 (5)
C6—C7—C10—C11	−175.8 (4)	C32—O3—C33—C34	−179.8 (3)
C6—C7—C10—C9	0.2 (6)	C35—O4—C34—C33	175.0 (3)
C8—C9—C10—C11	176.1 (4)	O3—C33—C34—O4	64.6 (4)
C14—C9—C10—C11	−4.0 (6)	C34—O4—C35—C36	179.2 (3)
C8—C9—C10—C7	−0.1 (6)	C37—O5—C36—C35	−175.9 (3)
C14—C9—C10—C7	179.8 (4)	O4—C35—C36—O5	−65.6 (4)
C7—C10—C11—C12	179.0 (4)	C36—O5—C37—C38	177.8 (3)
C9—C10—C11—C12	3.0 (6)	C39—O6—C38—C37	−176.4 (3)
C10—C11—C12—C13	0.4 (6)	O5—C37—C38—O6	65.3 (4)
C11—C12—C13—C14	−2.9 (6)	C38—O6—C39—C40	−170.6 (3)
C12—C13—C14—C9	1.8 (7)	C29—O1—C40—C39	177.1 (3)
C8—C9—C14—C13	−178.4 (4)	O6—C39—C40—O1	−72.0 (4)
C10—C9—C14—C13	1.7 (6)	C44—O7—C41—C42	−18.9 (6)
C15—C16—C17—C18	2.5 (6)	O7—C41—C42—C43	31.5 (6)
C20—C15—C16—C17	30.8 (6)	C41—C42—C43—C44	−31.8 (6)
C16—C17—C18—C19	−34.3 (6)	C41—O7—C44—C43	−2.3 (6)
Co1—C15—C16—C17	−58.9 (4)	C42—C43—C44—O7	22.0 (6)
Co1—C16—C17—C18	−58.6 (4)	C48—O8—C45—C46	−32.4 (8)
C15—C16—C17—Co1	61.1 (3)	O8—C45—C46—C47	31.7 (9)
C16—C17—C18—Co1	55.5 (3)	C45—C46—C47—C48	−17.0 (8)
C20—C15—C16—Co1	89.7 (3)	C45—O8—C48—C47	20.0 (8)
Co1—C17—C18—C19	−89.8 (4)	C46—C47—C48—O8	−2.1 (8)
C16—C15—C20—C19	−32.2 (5)	C52—O9—C49—C50	−34.7 (11)
C17—C18—C19—C20	31.7 (5)	O9—C49—C50—C51	17.2 (18)
C16—C15—C20—C21	150.7 (4)	C49—C50—C51—C52	7 (2)
C17—C18—C19—C22	−149.9 (4)	C50—C51—C52—O9	−29 (2)
C18—C19—C20—C15	1.5 (5)	C49—O9—C52—C51	39.4 (15)
C22—C19—C20—C21	0.4 (5)	C56—O10—C53—C54	−11 (3)
C18—C19—C20—C21	178.8 (3)	O10—C53—C54—C55	−13 (3)
C22—C19—C20—C15	−177.0 (3)	C53—C54—C55—C56	26 (2)
C19—C20—C21—C24	−1.2 (6)	C53—O10—C56—C55	31 (3)
C15—C20—C21—C24	175.8 (3)	C54—C55—C56—O10	−32 (2)
